# The burden and predictors of 30-day unplanned readmission in patients with acute liver failure: a national representative database study

**DOI:** 10.1186/s12876-024-03249-0

**Published:** 2024-05-03

**Authors:** Xianbin Xu, Kai Gong, Liang Hong, Xia Yu, Huilan Tu, Yan Lan, Junjie Yao, Shaoheng Ye, Haoda Weng, Zhiwei Li, Yu Shi, Jifang Sheng

**Affiliations:** 1 State Key Laboratory for Diagnosis and Treatment of Infectious Diseases, National Clinical Research Center for Infectious Diseases, Collaborative Innovation Center for Diagnosis and Treatment of Infectious Diseases, The First Affiliated Hospital, Zhejiang University School of Medicine, Hangzhou, 310000 Zhejiang China; 2 Department of Infectious Diseases, The Fourth Affiliated Hospital, Zhejiang University School of Medicine, Yiwu, 322000 Zhejiang China; 3 Division of Hepatobiliary and Pancreatic Surgery, Department of Surgery, The First Affiliated Hospital, Zhejiang University School of Medicine, Hangzhou, 310000 Zhejiang China

**Keywords:** ALF, Early readmission, Rehospitalization, National readmission database

## Abstract

**Background:**

Liver diseases were significant source of early readmission burden. This study aimed to evaluate the 30-day unplanned readmission rates, causes of readmissions, readmission costs, and predictors of readmission in patients with acute liver failure (ALF).

**Methods:**

Patients admitted for ALF from 2019 National Readmission Database were enrolled. Weighted multivariable logistic regression models were applied and based on Directed Acyclic Graphs. Incidence, causes, cost, and predictors of 30-day unplanned readmissions were identified.

**Results:**

A total of 3,281 patients with ALF were enrolled, of whom 600 (18.3%) were readmitted within 30 days. The mean time from discharge to early readmission was 12.6 days. The average hospital cost and charge of readmission were $19,629 and $86,228, respectively. The readmissions were mainly due to liver-related events (26.6%), followed by infection (20.9%). The predictive factors independently associated with readmissions were age, male sex (OR 1.227, 95% CI 1.023–1.472; *P* = 0.028), renal failure (OR 1.401, 95% CI 1.139–1.723; *P* = 0.001), diabetes with chronic complications (OR 1.327, 95% CI 1.053–1.672; *P* = 0.017), complicated hypertension (OR 1.436, 95% CI 1.111–1.857; *P* = 0.006), peritoneal drainage (OR 1.600, 95% CI 1.092–2.345; *P* = 0.016), etc.

**Conclusions:**

Patients with ALF are at relatively high risk of early readmission, which imposes a heavy medical and economic burden on society. We need to increase the emphasis placed on early readmission of patients with ALF and establish clinical strategies for their management.

**Supplementary Information:**

The online version contains supplementary material available at 10.1186/s12876-024-03249-0.

## Background

Acute liver failure (ALF) is the generic term used to describe the rapid development of severe liver dysfunction in the absence of preexisting liver diseases, mainly manifesting as coagulopathy and hepatic encephalopathy (HE) [1]. Paracetamol overdose is the primary aetiology of ALF in Western countries, while acute viral hepatitis is the leading cause in most countries in Asia and Africa [1]. ALF is a rare but life-threatening condition, imposing substantial health and economic burden on societies and healthcare systems. Despite the widespread application of liver transplantation (LT), the mortality of ALF remains as high as 33% [2]. In the United States, its estimated incidence ranges from 1 to 3,000 cases per million people annually [3]. In 2018, there were 25,089 emergency department visits and 26,480 adult hospital admissions with a principal diagnosis code for ALF [4].

Unplanned early (30-day) hospital readmission rate was frequently used to evaluate the quality of hospital care. Notably, several studies have consistently shown that the 30-day readmission rates of liver-related conditions led the way among digestive diseases [4, 5]. A latest study from the United States indicated that the early readmission rate of patients with liver disease reached as high as 31.4%, which was significantly higher than the 15% reported in 2018 [4, 6]. In recent years, detailed studies focused primarily on the early readmission burden of patients with cirrhosis. The overall 30-day readmission rate of cirrhotic patients was estimated ranging from 27 to 32% [7–9].

However, at present, there is a lack of research on the burden of readmission in patients with ALF, owing to the rarity of cases. On the other hand, in contrast to the natural progression of cirrhosis that leads to an ‘end-stage’ irreversible condition with recurrent episodes of complications, ALF is more fatal but potentially reversible in nature and thereby the expecting risk of readmission is low. This study sought to fill this gap by investigating the incidence rate, healthcare resource utilization, and predictors of short-term readmission in patients with ALF derived from a nation-wide database.

## Methods

### Data source

We performed this population-based study using the 2019 National Readmissions Database (NRD) that was developed for the Healthcare Cost and Utilization Project (HCUP) and sponsored by the Agency for Healthcare Research and Quality (AHRQ) [10]. The 2019 NRD is a large-sample size and all-payer inpatient database containing discharge data from 30 geographically diverse states, representing 61.8% of the total population and 60.4% of all hospitalizations in the United States. The 2019 NRD contains more than 100 clinical and nonclinical variables, including the diagnosis and procedure codes reported using the International Classification of Diseases, Tenth Revision, Clinical Modification/Procedure Coding System (ICD-10-CM/PCS coding system, version 2021.2). Data for the analyses were obtained from a public database, and no ethics committee approval or informed consent was waived.

### Inclusion and exclusion criteria

**Fig. 1:**
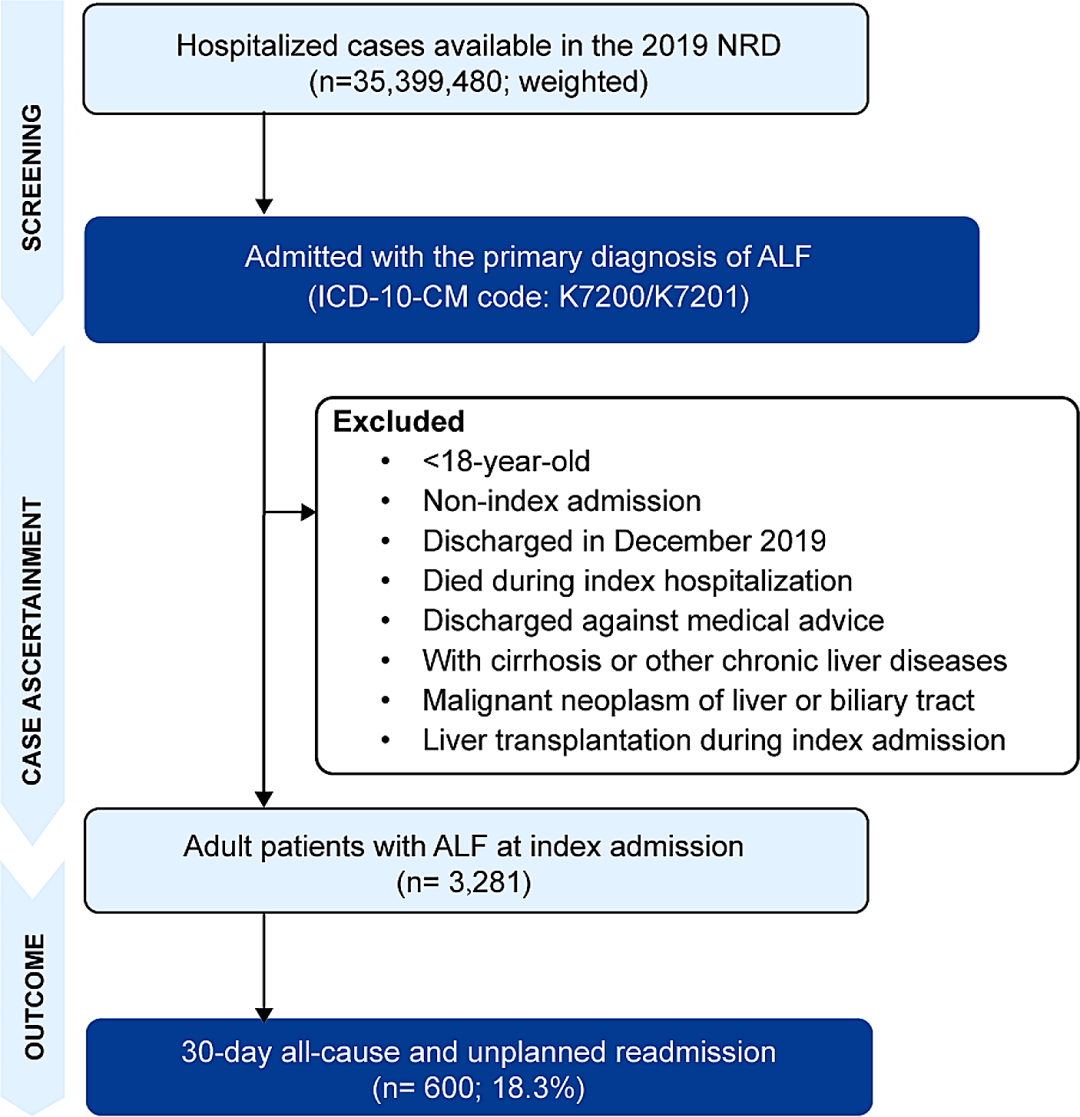
Flow chart of enrolled patients NRD, National Readmissions Database; ALF, acute liver failure

Using the 2019 NRD, we initially identified all adult patients (age ≥ 18 years) discharged with a primary diagnosis of ALF (ICD-10-CM K7200/K7201). The first admission in this period was considered as the index admission. Pairs of discharge records representing transfers were collapsed into a single record. For the acquisition of the 30-day follow-up data, patients discharged in December were excluded. Patients who died during the index admission or left against medical advice were excluded. Secondly, in accordance with previous studies, only those who without any diagnostic codes related to chronic liver diseases or cirrhosis were strictly considered as ALF and ultimately included in this study [11]. Cases received a liver transplant during index admission or diagnosed as malignant neoplasm of liver or biliary were removed from the cohort. Detailed definitions of diagnoses and procedures using ICD-10-CM codes are provided in Supplementary Table S1, and the patient flow chart is depicted in Fig. 1.

### Outcome

All-cause and unplanned readmission within 30 days after discharge was identified as the primary outcome for this study. Only the first readmission was included in the analyses. Reason for readmission was attributed to the primary discharge diagnoses of readmission which were classified into clinically meaningful categories based on Clinical Classifications Software Refined (CCSR) (Supplementary Table S2) [12]. Besides, in-hospital mortality during readmission and the burden on healthcare resources were evaluated. Based on Cost-to-Charge Ratio (CCR), the total charges for each discharge were converted into a cost estimate [13].

### Exposure variables

To explore risk factors for 30-day all-cause and unplanned readmission of patients with ALF, we gathered a comprehensive collection of potential exposures, covering socio-demographic status, medical data, and hospital characteristics (see Table 1). Median household income was estimated based on the ZIP Code of the patients and represented in a quartile classification: first quartile, $1 to $47,999; second quartile, $48,000 to $60,999; third quartile, $61,000 to $81,999; fourth quartile, >$82,000. Continues variables, including age and length of stay (LOS), were categorized into four groups according to the quartile distribution for subsequent analyses. Discharges with missing values (median household income, location of patients, payer, and elective index admission) were excluded (< 1.0% missing). ICD-10-CM/PCS codes for definitions of extrahepatic organ failures were shown in Supplementary Table S3.

**Table 1:** Characteristics of patients with ALF, stratified by 30-day all-cause and unplanned readmission status

Characteristics	Total *N* = 3,281	Readmission group *N* = 600	Non-readmission group *N* = 2,681	*P* value ^†^
**Age, years, median (IQR)**	61(47–72)	64(52–73)	60(46–71)	
19–47	835(25.4)	96(16.0)	739(27.6) ^*^	< 0.001
48–61	861(26.2)	166(27.7)	695(25.9)	
62–72	844(25.7)	178(29.7)	666(24.8) ^*^	
> 72	741(22.6)	160(26.7)	581(21.7) ^*^	
**Male**	1623(49.5)	323(53.8)	1300(48.5)	0.018
**Extrahepatic organ failure**				
Cardiovascular	107(3.3)	22(3.7)	85(3.2)	0.536
Respiratory	351(10.7)	57(9.5)	294(11.0)	0.294
Renal	1318(40.2)	308(51.3)	1010(37.7)	< 0.001
Brain	36(1.1)	6(1.0)	30(1.1)	0.800
**Comorbidities**				
Arthropathies	113(3.4)	16(2.7)	97(2.6)	0.251
Alcohol abuse	466(14.2)	82(13.7)	384(14.3)	0.680
Chronic pulmonary disease	614(18.7)	116(19.3)	498(18.6)	0.664
Depression	481(14.7)	68(11.3)	413(15.4)	0.011
Drug abuse	357(10.9)	61(10.2)	296(11.0)	0.534
Diabetes				
Without chronic complications	271(8.3)	52(8.7)	219(8.2)	0.689
With chronic complications	702(21.4)	185(30.8)	517(19.3)	< 0.001
Hypothyroidism	458(14.0)	100(16.7)	358(13.4)	0.033
Hypertension				
Uncomplicated	799(24.4)	134(22.3)	665(24.8)	0.202
Complicated	1022(31.1)	264(44.0)	758(28.3)	< 0.001
Obesity	491(15.0)	111(18.5)	380(14.2)	0.007
Peripheral vascular disease	144(4.4)	28(4.7)	116(4.3)	0.712
Lymphoma	39(1.2)	13(2.2)	26(1.0)	0.014
Solid malignancies	203(6.2)	47(7.8)	156(5.8)	0.064
**Elective index admission**	82(2.5)	16(2.7)	66(2.5)	0.770
**Procedures**				
Drainage of peritoneal cavity	166(5.1)	45(7.5)	121(4.5)	0.003
Gastrointestinal endoscopy	82(2.5)	13(2.2)	69(2.6)	0.564
Hemodialysis	244(7.4)	76(12.7)	168(6.3)	< 0.001
Transfusion of red blood cells	145(4.4)	37(6.2)	108(4.0)	0.021
Transfusion of plasma	88(2.7)	19(3.2)	69(2.6)	0.416
**LOS, days, median (IQR)**	4(3–8)	5(3–9)	4(2–8)	0.002
< 3	813(24.8)	121(20.2)	692(25.8) ^*^	0.010
3–4	887(27.0)	164(27.4)	723(27.0)	
5–8	880(26.8)	163(27.2)	717(26.7)	
> 8	700(21.3)	151(25.2)	549(20.5) ^*^	
***Social characteristics of patients***				
**Local residents** ^**‡**^	3077(93.8)	563(93.8)	2514(93.8)	0.954
**Location**				
Metropolitan counties	2695(82.1)	488(81.3)	2207(82.3)	0.195
Micropolitan	314(9.6)	52(8.7)	262(9.8)	
Not metropolitan or micropolitan counties	272(8.3)	60(10.0)	212(7.9)	
**Median household income**				
First quartile (lowest)	1028(31.3)	205(34.2)	823(30.7)	0.157
Second quartile	937(28.5)	160(26.7)	777(29.0)	
Third quartile	800(24.4)	153(25.5)	647(24.1)	
Fourth quartile (highest)	517(15.8)	82(13.7)	435(16.2)	
**Payer**				
Medicare	1608(49.0)	374(62.3)	1234(46.0) ^*^	< 0.001
Medicaid	618(18.8)	101(16.8)	517(19.3)	
Private insurance	778(23.7)	92(15.3)	686(25.6) ^*^	
Self-pay or other	278(8.5)	33(5.5)	245(9.1) ^*^	
***Hospital characteristics***				
**Hospital bedsize**				
Small	552(16.8)	97(16.2)	455(17.0)	0.798
Medium	805(24.5)	144(24.0)	661(24.7)	
Large	1924(58.6)	359(59.8)	1565(58.4)	
**Hospital location and teaching status**				
Metropolitan non-teaching	604(18.4)	100(16.7)	504(18.8)	0.369
Metropolitan teaching	2436(74.3)	450(75.1)	1986(74.1)	
Non-metropolitan	240(7.3)	49(8.2)	191(7.1)	
**Ownership of hospital**				
Government	412(12.6)	79(13.2)	333(12.4)	0.618
Private	2869(87.4)	521(86.8)	2348(87.6)	

Values are median (IQR) or n (%). Continues variables (Age and LOS) did not conform to a normal distribution and homogeneity (See the Supplementary Fig. S2). Comparisons were performed using χ² test for categorical variables and Wilcoxon rank-sum test for continues variables. ALF, acute liver failure; AIDS, acquired immune deficiency syndrome; LOS, length of stay

^†^*P* value for readmission vs. non-readmission group

^**‡**^ Patient located in the same state as the hospital

^*^ Significant (*P* < 0.05) difference between readmission and non-readmission group

### Statistical analyses

Categorical variables were presented as percentages, and continuous variables were expressed as mean (mean ± SD) or medians (median and IQR) values. The homogeneity and normality of continuous variables was checked using Levene’s test and Kolmogorov–Smirnov test, respectively (Supplementary Figure S1). Wilcoxon rank-sum test, independent t-test, and χ² test were appropriately used in this study. Weights were adjusted for all analyses to produce national estimates. In order to evaluate the exposure-outcome relationship, we constructed a multivariable logistic regression model for each exposure variable. Due to the subsumption relation between renal failure and hemodialysis, only renal failure was included in the logistic regression analysis. A sample size of 10–20 times the number of independent variables is ensured for multivariable regression. Directed acyclic graphs (DAG) were used to identify the potential confounders and intermediate variables between the exposures and outcome (Supplementary Figure S2). Potential confounders were maximally adjusted in the regression models, while the intermediate variables were excluded from the multivariable modeling (Supplementary Table S4). Results were reported as odds ratio (OR) and 95% confidence interval (CI). Analyses were performed using SPSS software (SPSS version 26.0; SPSS Inc). *P* < 0.05 (two-side) was statistically significant.

## Results

### Patient characteristics

A total of 3,281 (nationally weighted) patients were discharged alive with the primary diagnosis of ALF during the first 11 months of 2019. Among them, 600 (18.3%) were non-electively readmitted for all causes within 30 days. Table 1 shows the characteristics of study groups. Patients readmitted within 30 days tended to be older (median age 64 years vs. 60 years; *P* < 0.001). Men and women were equally distributed, but there were more males in the readmission group (53.8% vs. 48.5%, *P* = 0.018). The most common extrahepatic organ failure was renal failure (40.2%) and was more common in the readmission group (51.3% vs. 37.7%, *P* < 0.001). Of all the comorbidities investigated, diabetes with chronic complications (30.8% vs. 19.3%, *P* < 0.001), complicated hypertension (44.0% vs. 28.3%, *P* < 0.001), obesity (18.5% vs. 14.2%, *P* = 0.007), hypothyroidism (16.7% vs. 13.4%, *P* = 0.033), and lymphoma (2.2% vs. 1.0%, *P* = 0.014) were more common in patients with readmission, while depression was more frequently found in non-readmitted patients (15.4% vs. 11.3%, *P* = 0.011). Approximately 2% of the index admissions were elective, and the proportion were similar between the two groups. Besides, patients with early readmission were more likely to experience peritoneal drainage (7.5% vs. 4.5%, *P* = 0.003), hemodialysis (12.7% vs. 6.3%, *P* < 0.001), and red blood cell transfusion (6.2% vs. 4.0%, *P* = 0.021) during the index hospitalizations. The median hospital LOS was 5 days (IQR, 3–9 days) in readmission group and 4 days (IQR, 2–8 days) in non-readmission group (*P* = 0.002). In terms of social characteristics, patients with readmission had a higher proportion of Medicare (62.3% vs. 46.0%, *P* < 0.001), while patients’ location and median household income were not significantly different between the groups. Hospital characteristics, including bedsize, location and teaching status, and ownership were not significantly different between readmitted and non-readmitted patients. For the majority of cases, no underlying etiology could be identified by screening concomitant secondary diagnoses. However, in patients with possible etiology data, the most frequent etiology of ALF was drug or toxin induced liver injury. More details are shown in Table 2.

**Table 2:** Concomitant diagnoses to determine the etiology of acute liver failure (ALF)

Concomitant secondary diagnoses^†^	ICD-10-CM Code^‡^	ALF patients with diagnosis^§^		ALF patients with diagnosis and early readmission	
		*n* (weighted)	%of all ALF patients	*n* (weighted)	% of ALF patients with diagnosis
Drug/toxin induced liver diseases	K710, K7110, K7111, K712, K716, K718, K719; T36-T65	386	11.8	82	21.2
Acute viral hepatitis A	B150, B159	301	9.2	33	11.0
Acute viral hepatitis B	B160, B161, B162, B169	98	3.0	12	12.2
Acute viral hepatitis E	B172	139	4.3	0	0
Other acute viral hepatitis	B178, B179	139	4.3	25	18.0
Others^#^	B170, B251, B2790, B2791, B2792, B2799, E8301, I820, K754, K763	107	3.3	19	17.8

^†^Multiple assessments possible

^‡^ICD-10-CM: International Classification of Diseases, 10th Edition, Clinical Modification, v2021.2

^§^Total of 3,281 patients

^#^Acute viral hepatitis D (B170), cytomegaloviral hepatitis (B251), Infectious mononucleosis (B2790, B2791, B2792, B2799), wilson’s disease (E8301), budd-Chiari syndrome (I820), autoimmune hepatitis (K754), and infarction of liver (K763)

### Causes of readmissions

**Fig. 2:**
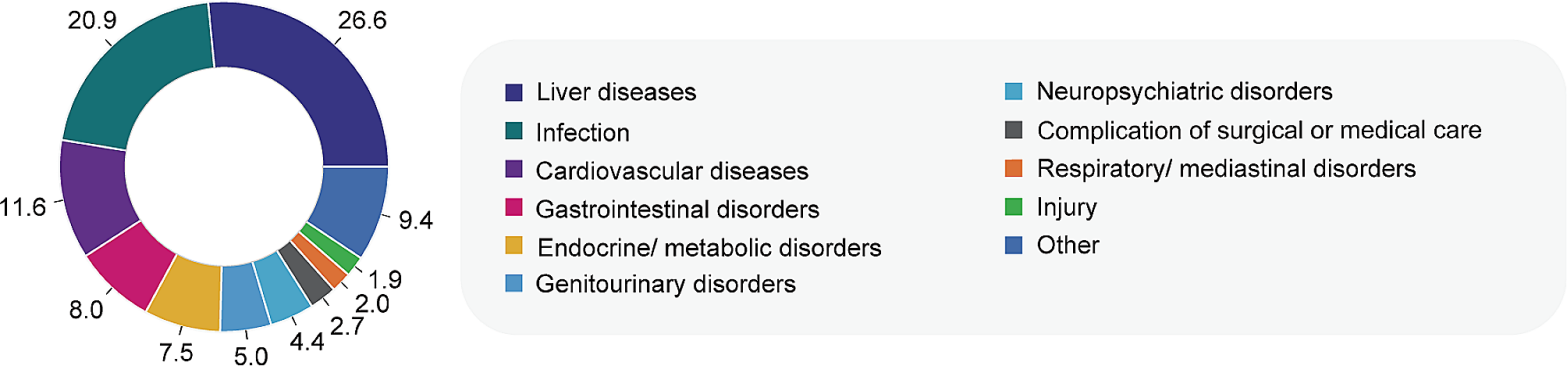
Distribution of primary discharge diagnostic categories of readmissions in patients with ALF Each diagnosis category is color-coded according to the right legend

Figure 2 lists the distribution of causes for 30-day unplanned readmission in patients with ALF. The most common cause of readmission was liver diseases (*n* = 159, 26.6%), followed by infections (*n* = 125, 20.9%) and cardiovascular diseases (*n* = 70, 11.6%). Of note, 60.4% (*n* = 96) of the 159 patients who readmitted due to liver diseases were still diagnosed with liver failure.

### Burden of healthcare resources

The mean time from discharge to all-cause readmission in patients with ALF was 12.6 days, which was close to 11.6 days for liver-related readmission and 13.0 days for infection-related readmission. The hospital LOS, cost, and charge of readmission were presented in Table 3. In contrast, patients readmitted for infection exhibit substantially longer hospital stays and heavier financial burdens. Strikingly, the mortality during rehospitalization was up to 23.0% in the infection-related group, which was significantly higher than that in the all-cause group (8.1%) and liver-related group (6.7%).

**Table 3:** Resource utilization of 30-day readmission in patients with ALF

Variables	All-cause (*n* = 600)	Liver-related (*n* = 159)	Infection-related (*n* = 125)
Mean time to readmission (days)	12.6	11.6	13.0
LOS of readmission (days)			
Mean	6.95	5.83	9.62
Total	4,167	930	1,207
Charge of readmission			
Mean ($)	86,228	75,795	150,181
Total (million)	52	12	19
Cost of readmission ^†^			
Mean ($)	19,629	17,234	30,532
Total (million)	12	3	4
Total died during readmission (%)	8.1	6.7	23.0

LOS, length of stay

^†^ The cost of inpatient care for a discharge is estimated by multiplying total charge with the corresponding cost-to-charge ratio

### Predictive factors of readmissions

The OR for readmission showed an increasing trend along with the increased age, and female patients were less likely than male patients to be readmitted (Table 4). ALF patients with renal failure had a higher risk of early readmission (OR1.401, 95% CI 1.139–1.723; *P* = 0.001). As regards the comorbidities, ALF patients who have diabetes with chronic complications (OR 1.327, 95% CI 1.053–1.672; *P* = 0.017) and complicated hypertension (OR 1.436, 95% CI 1.111–1.857; *P* = 0.006) had increased odds of 30-day all-cause readmission, while patients with depression (OR 0.720, 95% CI 0.541–0.958; *P* = 0.024) had a decreased odd of readmission. Patients undergoing peritoneal drainage (OR 1.600, 95% CI 1.092–2.345; *P* = 0.016) had a higher risk of 30-day all-cause readmission, but no significant associations of gastrointestinal endoscopy, red blood cell transfusion, and plasma transfusion with readmission were observed. Regarding primary expected payer, patients with private insurance (OR 0.523, 95% CI 0.390–0.701; *P* < 0.001) and self-pay/other (OR 0.578, 95% CI 0.375–0.891; *P* = 0.013) had lower odds of readmission than patients with Medicare insurance. Other social characteristics, including patients’ location, median household income, elective index admission, and hospital LOS had no effect on the outcome. And we did not find any significant association between hospital characteristics and 30-day rehospitalization (all *P* > 0.1).

**Table 4:** Predictive factors associated with 30-day all-cause and unplanned readmission of patients with acute liver failure (ALF)

Characteristics	OR (95% CI)	*P* value
**Age, years**		
19–47	1(ref)	
48–61	1.744(1.322–2.300)	< 0.001
62–72	1.849(1.399–2.443)	< 0.001
> 72	1.966(1.477–2.615)	< 0.001
**Male vs. Female**	1.227(1.023–1.472)	0.028
**Extrahepatic organ failure**		
Cardiovascular	1.003(0.598–1.680)	0.992
Respiratory	0.617(0.443–0.859)	0.004
Renal	1.401(1.139–1.723)	0.001
Brain	0.927(0.364–2.359)	0.873
**Comorbidities**		
Arthropathies	0.725(0.416–1.263)	0.256
Alcohol abuse	1.153(0.871–1.525)	0.320
Chronic pulmonary disease	0.917(0.721–1.167)	0.483
Depression	0.720(0.541–0.958)	0.024
Drug abuse	1.240(0.893–1.720)	0.199
Diabetes		
Non-diabetes	1(ref)	
Without chronic complications	1.105(0.787–1.552)	0.566
With chronic complications	1.327(1.053–1.672)	0.017
Hypothyroidism	1.230(0.949–1.594)	0.117
Hypertension		
Non- hypertension	1(ref)	
Uncomplicated	1.066(0.823–1.382)	0.628
Complicated	1.436(1.111–1.857)	0.006
Obesity	1.266(0.986–1.626)	0.064
Peripheral vascular disease	0.812(0.524–1.258)	0.352
Lymphoma	1.891(0.925–3.865)	0.081
Solid malignancies	1.301(0.910–1.859)	0.149
**Elective index admission**	1.201(0.669–2.155)	0.539
**Procedures**		
Drainage of peritoneal cavity	1.600(1.092–2.345)	0.016
Gastrointestinal endoscopy	0.621(0.334–1.154)	0.132
Transfusion of red blood cells	1.319(0.860–2.024)	0.205
Transfusion of plasma	1.000(0.563–1.777)	0.999
**LOS, days**		
< 3	1(ref)	
3–4	1.145(0.874–1.501)	0.326
5–8	1.015(0.769–1.341)	0.915
> 8	1.270(0.935–1.724)	0.126
**Local residents** ^**†**^	0.959(0.651–1.412)	0.832
**Location**		
Metropolitan counties	1(ref)	
Micropolitan	0.755(0.522–1.093)	0.137
Not metropolitan or micropolitan counties	1.027(0.719–1.468)	0.883
**Median household income**		
First quartile (lowest)	1(ref)	
Second quartile	0.861(0.676–1.098)	0.228
Third quartile	0.975(0.753–1.263)	0.851
Fourth quartile (highest)	0.811(0.595–1.105)	0.184
**Payer**		
Medicare	1(ref)	
Medicaid	0.743(0.549–1.007)	0.055
Private insurance	0.523(0.390–0.701)	< 0.001
Self-pay or other	0.578(0.375–0.891)	0.013
**Hospital bedsize**		
Small	1(ref)	
Medium	1.012(0.752–1.362)	0.940
Large	1.106(0.851–1.439)	0.451
**Hospital location and teaching status**		
Metropolitan non-teaching	1(ref)	
Metropolitan teaching	1.154(0.900–1.480)	0.258
Non-metropolitan	1.332(0.846–2.098)	0.216
**Ownership of hospital**		
Government	1(ref)	
Private	0.937(0.709–1.238)	0.647

Intermediate variables were excluded from the multivariable modeling, and confounders were maximally adjusted in the regression models. For the association of age with outcome, sex, extrahepatic organ failure, elective index admission, procedures, and hospital characteristics were adjusted. For the association of sex with outcome, age, extrahepatic organ failure, social characteristics of patients, elective index admission, procedures, and hospital characteristics. When exploring the associations of extrahepatic organ failure, comorbidities, and procedures with outcome, all variables except for LOS were included. All variables were adjusted for the association of hospital characteristics, elective index admission, LOS, and social characteristics of patients with outcome. OR, odds ratio; CI, confidence interval; LOS, length of stay

^†^ Patient located in the same state as the hospital

## Discussion

Early unplanned hospital readmission is a common and costly health-care issue [14]. In order to reduce avoidable readmissions, the Affordable Care Act established the Hospital Readmissions Reduction Program in 2012, which reduced the Centers for Medicare and Medicaid Services payments to hospitals with excess readmissions for specific conditions or procedures, including acute myocardial infarction, chronic obstructive pulmonary disease, heart failure, etc. [15]. However, liver diseases, such as liver failure and cirrhosis, have yet to be included in the program. In the past few decades, researchers have focused primarily on the early readmission in patients with cirrhosis, but relatively little is known about the readmission of patients with ALF. Our results revealed that 18.3% of patients with ALF readmitted for various reasons within 30 days after discharged, which was higher than the reported national average readmission rates for other conditions (11.6%) [16]. Not only that, our data also showed that early readmission of patients with ALF imposed both a significant burden on the healthcare system and the families. These unexpected findings recognized a significant clinical unmet in the management of ALF.

Infection is a non-negligible cause for readmission in patients with ALF. In our cohort, up to 20.9% of ALF patients were readmitted with a primary discharge diagnosis related to infection. These patients required longer hospital stays and higher medical expenditures. Of note, these patients had a high risk for in-hospital death, reaching 23.0%. Infection in patients with ALF is a frequent trigger of cerebral oedema, multisystem organ failure and delisting of transplantation [1]. Sepsis imposes undesirable effects on liver transplantation and increases the mortality rate of ALF by 10 to 52% [17]. There has been a plenty of evidence indicating a defective antimicrobial immunity in patients with ALF, which is associated with increased susceptibility to infection [18–20]. For example, low HLA-DR expression on monocytes of ALF patients due to the “spill-over” compensatory anti-inflammatory response, results in persistent functional monocyte deactivation [18]. Phagocytic, cytotoxic, and intracellular killing capacities of neutrophils and both classical and alternative complement pathways are also impaired in ALF [19, 20]. Previously, the U.S. Acute Liver Failure Study Group recommended empirical broad-spectrum antibiotics should be administered to ALF patient with progressive or advanced HE, with the presence of systemic inflammatory response syndrome, or listed for LT [21]. A multi-center retrospective cohort study indicated that antimicrobial prophylaxis does not decrease the incidence of bloodstream infection and mortality in patients with ALF [22]. However, the choice of antibiotics and time for prophylaxis initiation may vary among different medical centers, and other strategies for preventing bloodstream infections, such as sterile tubing maintenance, were not factored into assessments in this study [17]. And the impact of infections or antimicrobial prophylaxis on the early readmission of patients with ALF was not evaluated.

Chronic comorbidities were associated with early admission of patients with ALF. Our study demonstrated that diabetes with chronic complications, and complicated hypertension were associated with increased risk of readmission. A previous study reported that patients with cirrhosis who were readmitted within 30 days had more comorbidities than those who were not readmitted, and the presence of comorbidities was one of the predictors of readmission [7]. Not only that, another study in patients with advanced liver diseases concluded that diabetes increased the risk of 30-day readmission by 78% [23]. However, our study was unable to investigate the interplay between comorbidities and ALF, which may offer a precise reason for readmission. Nevertheless, our findings indicated the importance of management of comorbidities after discharge in preventing avoidable early readmission in patients with ALF.

Additionally, it was noted that 5.1% of patients experienced peritoneal drainage during the index admission, and the readmission risk for these patients increased approximately 1.6-fold. On one hand, overt ascites is more frequent in patients with subacute type of ALF, which have a consistently worse outcome and a more prolonged course than those in whom the illness has a more rapid onset. On the other hand, the presence of overt ascites is linked to a variety of complications such as spontaneous bacterial peritonitis, intestinal dysfunction, AKI, and even diaphragm dysfunction [24]. Hence, patients complicated with overt ascites during hospitalization were at increased risk of readmission and long-term management and outpatient follow-up are particularly needed for patients with ascites.

AKI in patients with ALF is very common. It is reported that 70% of patients with ALF developed AKI, and 30% needed renal replacement therapy (RRT) [25]. Typically, RRT was mainly applied in patients with uremia, volume overload, and hyperkalaemia. Whereas for patients with ALF, guidelines recommended that early RRT should be applied in patients with hyperammonaemia or progressive HE. Based on the ICD-10-PCS codes, we investigated the association between the use of hemodialysis and readmission in patients with ALF. In our cohort, 40.2% cases had a combination of renal failure and 7.4% of patients underwent hemodialysis during the index admission. The readmission risk of these patients increased by 40% compared with those who did not have renal failure. Collectively, the results indicated the impact of the episodes of extra-hepatic organ dysfunction/failure on the risk of readmission in patients with ALF. However, the specific impact and the preventive measures needed to be further investigated.

Other risk factors associated with 30-day readmission of patients with ALF confirmed in this study include age, gander, payer, etc. We noticed that the risk of readmission was consistently increasing with age, and men were high-risk population. In addition, patients with private insurance or self-pay have a lower readmission risk than those with Medicare insurance. These results may provide enlightenment for quality improvement in these special populations. Therefore, in order to reduce the socio-economic burden carried by readmission of patients with ALF, we should pay more attention to these special populations and take targeted and explicit guidance and assistance.

Although this was a large, retrospective, and multicenter study based on a national healthcare system with complete database records, several limitations remained. First, several methodological factors should be considered. In our study, diagnoses were ascertained through ICD-10-CM codes, but the inaccuracy of diagnoses may be present. Therefore, we conducted an extensive review of procedural and diagnostic codes for each case to achieve the most accurate identification of patients. In addition, although the etiology of patients with ALF is difficult to determine, we performed extensive screening for concomitant diagnoses related to possible etiological explanations in patients with ALF. Second, the database cannot identify patients who were readmitted or transferred between the states, as each State Inpatient Databases uses different codes to track patients. Third, the NRD also lacks specific clinical variables such as laboratory data, radiological features, and pathologic findings. Therefore, more informative predictors of readmission may be missed. However, this study represented the first effort to explore the 30-day unplanned readmission of patients with ALF. More researches are needed to strengthen the evidences.

## Conclusion

Patients with ALF has a high rate of early readmission, which inflicts a heavy medical and economic burden on society. Clinicians should raise the awareness of early readmission in patients with ALF and strengthen the management of complications. Strategies that help reduce financial consequences should continue to be explored.

### Electronic supplementary material

Below is the link to the electronic supplementary material.

Supplementary Material 1

## Data Availability

The data that support the findings of this study are openly available in Healthcare Cost and Utilization Project (HCUP) Nationwide Readmissions Database (NRD). https://www.hcup-us.ahrq.gov.

## Abbreviations

AHRQ: Agency for Healthcare Research and Quality
AKI: Acute kidney injury
ALF: Acute liver failure
AMA: Against medical advice
CI: Confidence interval
CLD: Chronic liver diseases
CMS: Centers for Medicare and Medicaid Services
DAG: Directed acyclic graph
HE: Hepatic encephalopathy
HCUP: Healthcare Cost and Utilization Project
HRRP: Hospital Readmissions Reduction Program
ICD-10-CM/PCS: International Classification of Diseases, Tenth Revision, Clinical; Modification/Procedure
LOS: Length of stay
LT: Liver transplantation
NRD: National Readmissions Database
OR: Odds ratio
RRT: Renal replacement therapy
