# Supplementary material for: The burden and predictors of 30-day unplanned readmission in patients with acute liver failure: a national representative database study
Source: BMC Gastroenterol. 2024 May 3;24:153. doi: 10.1186/s12876-024-03249-0 (PMC11067096; doi:10.1186/s12876-024-03249-0)
Supplement: Supplementary file 1 — Supplementary Material 1 [file 12876_2024_3249_MOESM1_ESM.docx]

**Supplementary Materials**

The burden and predictors of 30‐day unplanned readmission in patients with acute liver failure: a national representative database study

Xianbin Xu, Kai Gong, Liang Hong, Xia Yu, Huilan Tu, Yan Lan, Junjie Yao, Shaoheng Ye, Haoda Weng, Zhiwei Li, Yu Shi and Jifang Sheng

**Contents**

[Table S1. ICD-10-CM/PCS codes for definitions of diagnoses and procedures. 1](#_Toc161398859)

[Table S2. Clinical Classifications Software Refined (CCSR) for causes of readmission. 3](#_Toc161398860)

[Table S3. ICD-10-CM/PCS codes for definitions of extrahepatic organ failures. 11](#_Toc161398861)

[Table S4. Intermediate variables between the exposures and outcomes. 12](#_Toc161398862)

[Figure S1. Test of homogeneity and normality. 13](#_Toc161398863)

[Figure S2. Directed acyclic graph (DAG) for the identification of potential confounders and intermediate variables between the exposures and outcome, with age as an example. 14](#_Toc161398864)

Table S1. ICD-10-CM/PCS codes for definitions of diagnoses and procedures.

| Diagnoses/procedures | ICD-10-CM/ PCS Codes |
| --- | --- |
| Cirrhosis | |
| Alcoholic cirrhosis | K7030, K7031 |
| Non-alcoholic cirrhosis | K717, K743, K744, K745, K7460, K7469, P7881 |
| Other chronic liver diseases | |
| Alcoholic liver disease | K7010, K7011, K702, K7040, K7041, K709 |
| Chronic viral hepatitis | B180, B181, B182, B188, B189 |
| Other chronic hepatitis | K730, K731, K732, K738, K739 |
| Hepatic fibrosis or/and sclerosis | K740, K741, K742 |
| Other chronic disorders of the liver | K713, K714, K7150, K7151, K7210, K7211, K752, K753, K7581, K7589, K759, K760, K761, K764, K765, K7689, K769, K77 |
| Malignant neoplasm of liver or biliary | |
| Malignant neoplasm of liver | C220, C222, C223, C224, C227, C228, C229 |
| Malignant neoplasm of intrahepatic bile duct | C221 |
| Malignant neoplasm of gallbladder | C23 |
| Malignant neoplasm of other and unspecified parts of biliary tract | C240, C248, C241, C249 |
| Secondary malignant neoplasm of liver and intrahepatic bile duct | C787 |
| Procedures | |
| Liver transplant | 0FY00Z0, 0FY00Z1, 0FY00Z2 |
| Drainage of Peritoneal Cavity | 0W9G30Z, 0W9G3ZX, 0W9G3ZZ |
| Hemodialysis | 5A1D00Z, 5A1D60Z, 5A1D70Z, 5A1D80Z, 5A1D90Z |
| Gastrointestinal system endoscopy  (Upper intestinal tract) | 0DJ08ZZ, 0DJ68ZZ, 0DJD8ZZ, 0WJP8ZZ |
| Transfusion of red blood cells | 30233N0, 30233N1 |
| Transfusion of plasma | 30233K0, 30233K1, 30233L0, 30233L1 |

ICD-10-CM/PCS: International Classification of Diseases, 10th Edition, Clinical Modification/Procedure Coding System, v2021.2.

Table S2. Clinical Classifications Software Refined (CCSR) for causes of readmission.

| Categories of readmissions | CCSR Description, v2021.2 |
| --- | --- |
| Liver diseases | DIG018 Hepatic failure |
|  | DIG019 Other specified and unspecified liver disease |
|  | DIG023 Noninfectious hepatitis |
|  | INF007 Hepatitis |
| Gastrointestinal disorders | DIG004 Esophageal disorders |
|  | DIG005 Gastroduodenal ulcer |
|  | DIG006 Gastrointestinal and biliary perforation |
|  | DIG007 Gastritis and duodenitis |
|  | DIG008 Other specified and unspecified disorders of stomach and duodenum |
|  | DIG009 Appendicitis and other appendiceal conditions |
|  | DIG010 Abdominal hernia |
|  | DIG011 Regional enteritis and ulcerative colitis |
|  | DIG012 Intestinal obstruction and ileus |
|  | DIG013 Diverticulosis and diverticulitis |
|  | DIG014 Hemorrhoids |
|  | DIG015 Anal and rectal conditions |
|  | DIG016 Peritonitis and intra-abdominal abscess |
|  | DIG017 Biliary tract disease |
|  | DIG020 Pancreatic disorders (excluding diabetes) |
|  | DIG021 Gastrointestinal hemorrhage |
|  | DIG022 Noninfectious gastroenteritis |
|  | DIG024 Postprocedural or postoperative digestive system complication |
|  | DIG025 Other specified and unspecified gastrointestinal disorders |
| Infection | INF001 Tuberculosis |
|  | INF002 Septicemia |
|  | INF003 Bacterial infections |
|  | INF004 Fungal infections |
|  | INF005 Foodborne intoxications |
|  | INF006 HIV infection |
|  | INF008 Viral infection |
|  | INF009 Parasitic, other specified and unspecified infections |
|  | INF010 Sexually transmitted infections (excluding HIV and hepatitis) |
|  | INF011 Sequela of specified infectious disease conditions |
|  | INF012 Coronavirus disease – 2019 (COVID-19) |
|  | MUS001 Infective arthritis |
|  | MUS002 Osteomyelitis |
|  | NVS001 Meningitis |
|  | NVS002 Encephalitis |
|  | NVS003 Other specified CNS infection and poliomyelitis |
|  | NVS014 CNS abscess |
|  | RSP001 Sinusitis |
|  | RSP002 Pneumonia (except that caused by tuberculosis) |
|  | RSP003 Influenza |
|  | RSP004 Acute and chronic tonsillitis |
|  | RSP005 Acute bronchitis |
|  | RSP006 Other specified upper respiratory infections |
|  | RSP010 Aspiration pneumonitis |
|  | DIG001 Intestinal infection |
|  | SKN001 Skin and subcutaneous tissue infections |
|  | SKN002 Other specified inflammatory condition of skin |
|  | GEN004 Urinary tract infections |
| Genitourinary disorders | GEN001 Nephritis; nephrosis; renal sclerosis |
|  | GEN002 Acute and unspecified renal failure |
|  | GEN003 Chronic kidney disease |
|  | GEN005 Calculus of urinary tract |
|  | GEN006 Other specified and unspecified diseases of kidney and ureters |
|  | GEN007 Other specified and unspecified diseases of bladder and urethra |
|  | GEN008 Urinary incontinence |
|  | GEN009 Hematuria |
|  | GEN010 Proteinuria |
|  | GEN011 Vesicoureteral reflux |
|  | GEN012 Hyperplasia of prostate |
|  | GEN013 Inflammatory conditions of male genital organs |
|  | GEN014 Erectile dysfunction |
|  | GEN015 Male infertility |
|  | GEN016 Other specified male genital disorders |
|  | GEN017 Nonmalignant breast conditions |
|  | GEN018 Inflammatory diseases of female pelvic organs |
|  | GEN019 Endometriosis |
|  | GEN020 Prolapse of female genital organs |
|  | GEN021 Menstrual disorders |
|  | GEN022 Benign ovarian cyst |
|  | GEN023 Menopausal disorders |
|  | GEN024 Female infertility |
|  | GEN025 Other specified female genital disorders |
|  | GEN026 Postprocedural or postoperative genitourinary system complication |
| Cardiovascular diseases | CIR001 Chronic rheumatic heart disease |
|  | CIR002 Acute rheumatic heart disease |
|  | CIR003 Nonrheumatic and unspecified valve disorders |
|  | CIR004 Endocarditis and endocardial disease |
|  | CIR005 Myocarditis and cardiomyopathy |
|  | CIR006 Pericarditis and pericardial disease |
|  | CIR007 Essential hypertension |
|  | CIR008 Hypertension with complications and secondary hypertension |
|  | CIR009 Acute myocardial infarction |
|  | CIR010 Complications of acute myocardial infarction |
|  | CIR011 Coronary atherosclerosis and other heart disease |
|  | CIR012 Nonspecific chest pain |
|  | CIR013 Acute pulmonary embolism |
|  | CIR014 Pulmonary heart disease |
|  | CIR015 Other and ill-defined heart disease |
|  | CIR016 Conduction disorders |
|  | CIR017 Cardiac dysrhythmias |
|  | CIR018 Cardiac arrest and ventricular fibrillation |
|  | CIR019 Heart failure |
| Respiratory/ Mediastinal disorders | RSP008 Chronic obstructive pulmonary disease and bronchiectasis |
|  | RSP009 Asthma |
|  | RSP011 Pleurisy, pleural effusion and pulmonary collapse |
|  | RSP012 Respiratory failure; insufficiency; arrest |
|  | RSP013 Lung disease due to external agents |
|  | RSP014 Pneumothorax |
|  | RSP015 Mediastinal disorders |
|  | RSP016 Other specified and unspecified lower respiratory disease |
|  | RSP017 Postprocedural or postoperative respiratory system complication |
| Endocrine/metabolic disorders | END001 Thyroid disorders |
|  | END002 Diabetes mellitus without complication |
|  | END003 Diabetes mellitus with complication |
|  | END004 Diabetes mellitus, Type 1 |
|  | END005 Diabetes mellitus, Type 2 |
|  | END006 Diabetes mellitus, due to underlying condition, drug or chemical induced, or other specified type |
|  | END007 Nutritional deficiencies |
|  | END008 Malnutrition |
|  | END009 Obesity |
|  | END010 Disorders of lipid metabolism |
|  | END011 Fluid and electrolyte disorders |
|  | END012 Cystic fibrosis |
|  | END013 Pituitary disorders |
|  | END014 Postprocedural or postoperative endocrine or metabolic complication |
|  | END015 Other specified and unspecified endocrine disorders |
|  | END016 Other specified and unspecified nutritional and metabolic disorders |
|  | END017 Sequela of malnutrition and other nutritional deficiencies |
| Injury | INJ001 Fracture of head and neck |
|  | INJ002 Fracture of the spine and back |
|  | INJ003 Fracture of torso |
|  | INJ004 Fracture of the upper limb |
|  | INJ005 Fracture of the lower limb (except hip) |
|  | INJ006 Fracture of the neck of the femur (hip) |
|  | INJ007 Dislocations |
|  | INJ008 Traumatic brain injury (TBI); concussion |
|  | INJ009 Spinal cord injury (SCI) |
|  | INJ010 Internal organ injury |
|  | INJ011 Open wounds of head and neck |
|  | INJ012 Open wounds to limbs |
|  | INJ013 Open wounds of trunk |
|  | INJ014 Amputation of a limb |
|  | INJ015 Amputation of other body parts |
|  | INJ016 Injury to blood vessels |
|  | INJ017 Superficial injury; contusion |
|  | INJ018 Crushing injury |
|  | INJ019 Burn and corrosion |
|  | INJ020 Effect of foreign body entering opening |
|  | INJ021 Effect of other external causes |
| Complication of surgical or medical care | INJ033 Complication of cardiovascular device, implant or graft |
|  | INJ034 Complication of genitourinary device, implant or graft, |
|  | INJ035 Complication of internal orthopedic device or implant |
|  | INJ036 Complication of transplanted organs or tissue |
|  | INJ037 Complication of other surgical or medical care, injury |
| Neuropsychiatric disorders | MBD001 Schizophrenia spectrum and other psychotic disorders |
|  | MBD002 Depressive disorders |
|  | MBD003 Bipolar and related disorders |
|  | MBD004 Other specified and unspecified mood disorders |
|  | MBD005 Anxiety and fear-related disorders |
|  | MBD006 Obsessive-compulsive and related disorders |
|  | MBD007 Trauma- and stressor-related disorders |
|  | MBD008 Disruptive, impulse-control and conduct disorders |
|  | MBD009 Personality disorders |
|  | MBD012 Suicidal ideation/attempt/intentional self-harm |
|  | MBD013 Miscellaneous mental and behavioral disorders/conditions |
|  | MBD014 Neurodevelopmental disorders |
|  | MBD017 Alcohol-related disorders |
|  | MBD018 Opioid-related disorders |
|  | MBD019 Cannabis-related disorders |
|  | MBD020 Sedative-related disorders |
|  | MBD021 Stimulant-related disorders |
|  | MBD022 Hallucinogen-related disorders |
|  | MBD023 Inhalant-related disorders |
|  | MBD024 Tobacco-related disorders |
|  | NVS004 Parkinson`s disease |
|  | NVS005 Multiple sclerosis |
|  | NVS006 Other specified hereditary and degenerative nervous system conditions |
|  | NVS007 Cerebral palsy |
|  | NVS008 Paralysis (other than cerebral palsy) |
|  | NVS009 Epilepsy; convulsions |
|  | NVS010 Headache; including migraine |
|  | NVS011 Neurocognitive disorders |
|  | NVS012 Transient cerebral ischemia |
|  | NVS013 Coma; stupor; and brain damage |
|  | NVS015 Polyneuropathies |
|  | NVS016 Sleep wake disorders |
|  | NVS017 Nerve and nerve root disorders |
|  | NVS018 Myopathies |
|  | NVS019 Nervous system pain and pain syndromes |
|  | NVS020 Other specified nervous system disorders |

Table S3. ICD-10-CM/PCS codes for definitions of extrahepatic organ failures.

| Diagnoses | ICD-10-CM/PCS Codes |
| --- | --- |
| Cardiovascular failure | |
| Central venous pressure | 4A040B0, 4A043B0 |
| Pulmonary arterial pressure monitoring | 4A13353, 4A133B3, 4A133J3 |
| Arterial line | 4A130B1,4A033B1 |
| Septic shock | R6521 |
| Severe sepsis | R6520 |
| Pulmonary failure | |
| Mechanical ventilation | 5A1935Z, 5A1945Z, 5A1955Z |
| Acute respiratory failure | J9600, J9601, J9602 |
| Renal failure | |
| Hemodialysis | 5A1D00Z, 5A1D60Z, 5A1D70Z, 5A1D80Z, 5A1D90Z |
| Acute kidney failure | N170, N171, N172, N178, N179 |
| Brain failure | |
| Liver failure with coma | K7201 |

ICD-10-CM/PCS: International Classification of Diseases, 10^th^ Edition, Clinical Modification/Procedure Coding System, v2021.2.

Table S4. Intermediate variables between the exposures and outcomes.

| Exposure variables | Intermediate variables |
| --- | --- |
| Age | Comorbidities, Social characteristics of patients, LOS |
| Sex | Comorbidities, LOS |
| Extrahepatic organ failure | LOS |
| Comorbidities | LOS |
| Procedures | LOS |
| Hospital characteristics^b^, elective index admission, LOS, social characteristics of patients^a^ | / |

Potential intermediate variables and confounders were selected based on direct acyclic graphs (DAG, Supplementary Figure S2). LOS, length of stay.

^a^ Including local residents (patient located in the same state as the hospital), location, median household income, and payer.

^b^ Including hospital bedsize, location and teaching status, and ownership.

Figure S1. Test of homogeneity and normality.

Tow variables (Age and Length of stay) were tested for homogeneity of variances by using Levene’s test for homogeneity of variances and for normality by using the Kolmogorov–Smirnov tests of normality and normal Q – Q plots.


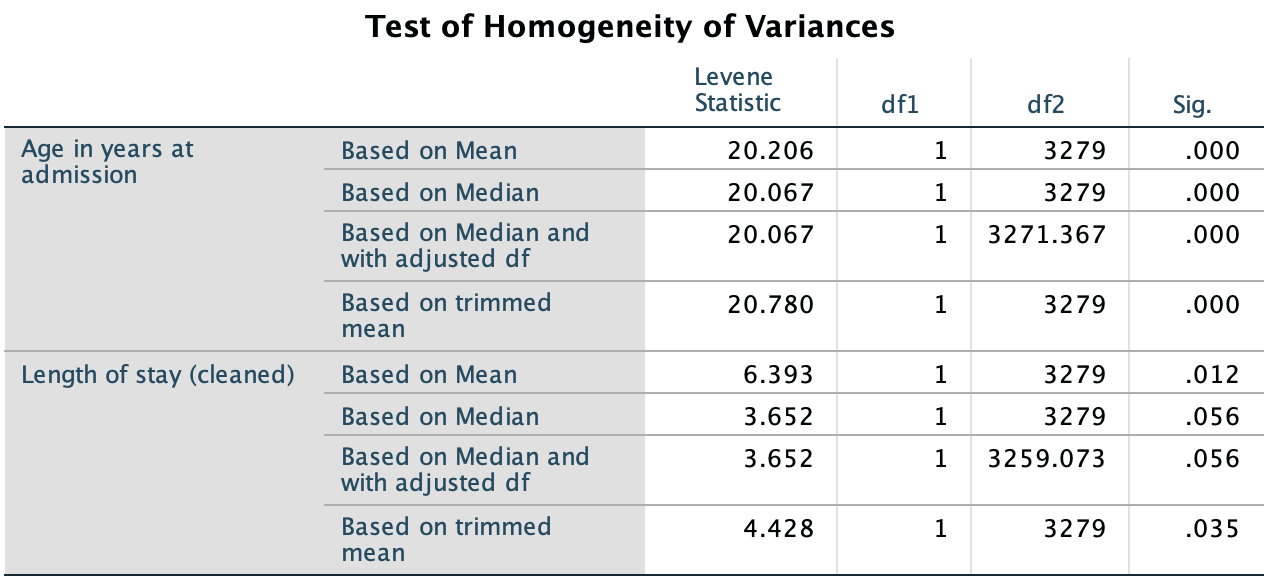

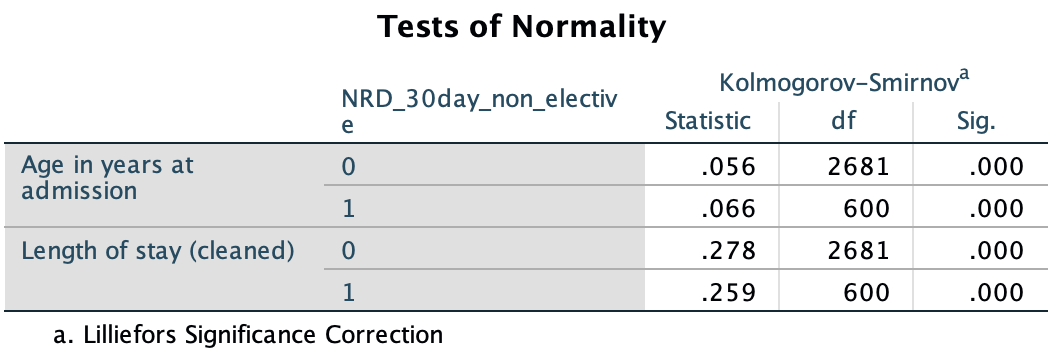

Figure S2. Directed acyclic graph (DAG) for the identification of potential confounders and intermediate variables between the exposures and outcome, with age as an example.


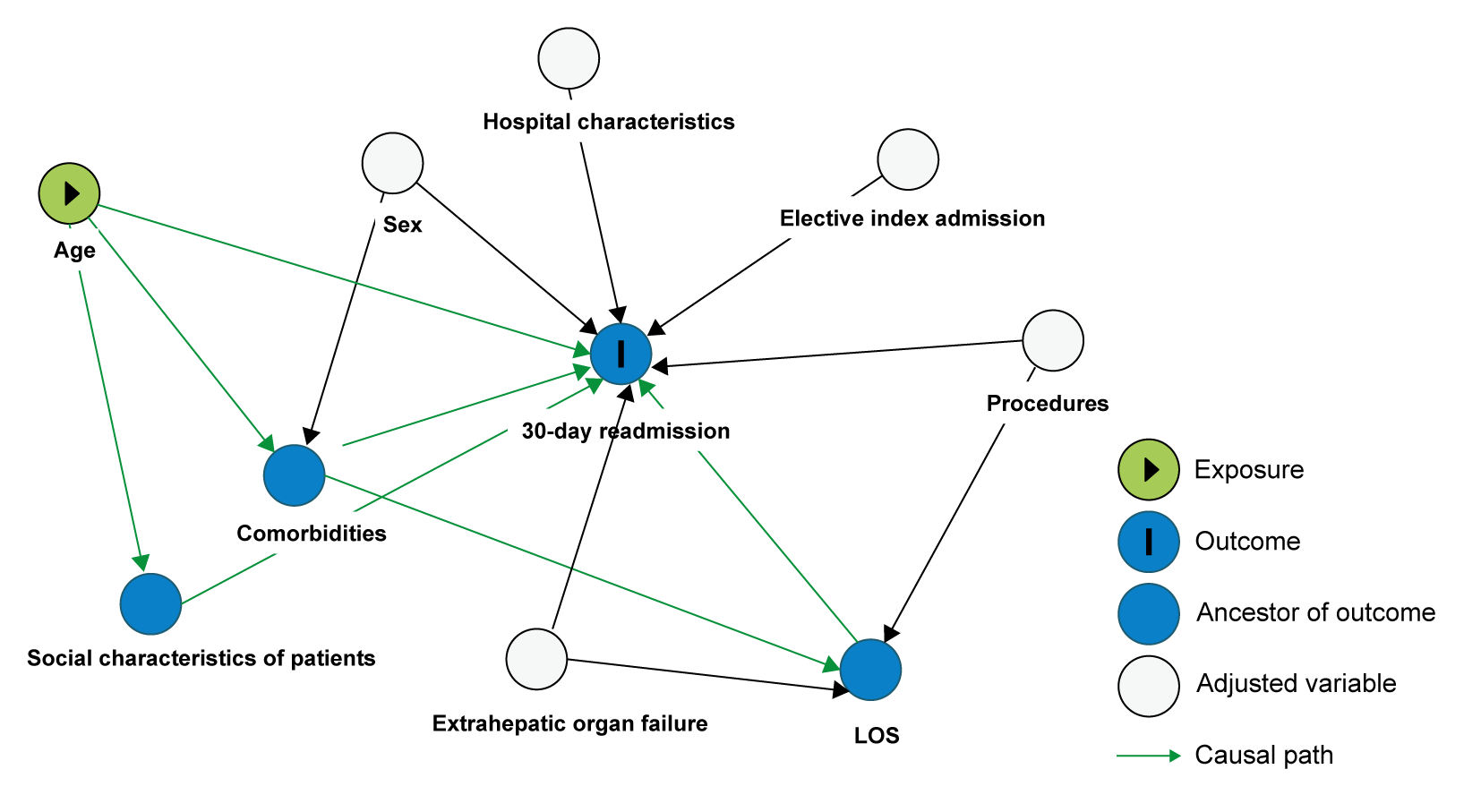


Potential confounders evaluated were sex, elective index admission, procedures, and hospital characteristics (bedsize, location and teaching status, and ownership). Intermediate variables, including comorbidities, social characteristics of patients (local residents, location, median household income, and payer), and LOS were excluded from the multivariable analysis. LOS, length of stay.
